# Satellitome Analysis in the Ladybird Beetle *Hippodamia variegata* (Coleoptera, Coccinellidae)

**DOI:** 10.3390/genes11070783

**Published:** 2020-07-13

**Authors:** Pablo Mora, Jesús Vela, Francisco J. Ruiz-Ruano, Areli Ruiz-Mena, Eugenia E. Montiel, Teresa Palomeque, Pedro Lorite

**Affiliations:** 1 Department of Experimental Biology, Genetic Area, University of Jaén, 23071 Jaén, Spain; pmora@ujaen.es (P.M.); jvela@ujaen.es (J.V.); armena@ujaen.es (A.R.-M.); emontiel@ujaen.es (E.E.M.); tpalome@ujaen.es (T.P.); 2 Department of Organismal Biology, Systematic Biology, Evolutionary Biology Centre, Uppsala University, SE-752 36 Uppsala, Sweden; francisco.ruiz-ruano@ebc.uu.se

**Keywords:** Coleoptera, Coccinellidae, *Hippodamia variegata*, ladybird beetle, RepeatExplorer, satellite DNA, satellitome, in situ hybridization

## Abstract

*Hippodamia variegata* is one of the most commercialized ladybirds used for the biological control of aphid pest species in many economically important crops. This species is the first Coccinellidae whose satellitome has been studied by applying new sequencing technologies and bioinformatics tools. We found that 47% of the *H. variegata* genome is composed of repeated sequences. We identified 30 satellite DNA (satDNA) families with a median intragenomic divergence of 5.75% and A+T content between 45.6% and 74.7%. This species shows satDNA families with highly variable sizes although the most common size is 100–200 bp. However, we highlight the existence of a satDNA family with a repeat unit of 2 kb, the largest repeat unit described in Coleoptera. PCR amplifications for fluorescence in situ hybridization (FISH) probe generation were performed for the four most abundant satDNA families. FISH with the most abundant satDNA family as a probe shows its pericentromeric location on all chromosomes. This location is coincident with the heterochromatin revealed by C-banding and DAPI staining, also analyzed in this work. Hybridization signals for other satDNA families were located only on certain bivalents and the X chromosome. These satDNAs could be very useful as chromosomal markers due to their reduced location.

## 1. Introduction

Eukaryotic genomes are composed of single copy sequences and repetitive sequences that are mainly responsible for the structure, size and genome diversity [1]. The repeated DNA is composed of transposable elements and tandem repetitions [2]. The most significant portion in a eukaryotic genome is composed of repetitive DNA sequences tandemly repeated among the genome, known as satellite DNA (satDNA), and discovered in cesium-chloride density gradients [3]. They are constituted by highly repeated sequences with a repeat unit or monomer with variable size. Generally, the mean size of the monomer among satDNA families can vary between 100 and 1000 bp, although it changes widely [4]. These repeated sequences are typically found in the heterochromatin [5], being part of chromosome structures, such as telomeres [6] and pericentromeric and centromeric regions [1]. The amount of satDNA present in a eukaryotic genome depends on the organism and in insects it can be up to the 50% of the total DNA [7,8]. The pericentromeric preferential location of the repetitive DNA allows creation of a particular nuclear architecture that may determine unequal transmission or expression properties, even with the same coding sequences [9]. Because of these properties, the repetitive components in the genome can play an important role in the evolutionary diversification and divergence of the species as well as increasing the genome size [10,11]. The evolution rate of repetitive sequences is very fast and they can vary in nucleotide composition, chromosomal distribution or genome representation. These factors can affect the genome structure or they can affect the genome size between species [12]. Sometimes, the satDNA is chromosome-, species- or genome-specific in many species and may cause a large variation between close species [13,14]. Normally, the satDNA is A+T rich, which is why the satDNA has been related to the curvature and the condensation of the chromatin [15].

Satellite DNA has been historically called “junk”, “parasite” or “selfish” DNA because its functions are not still well elucidated and it has been thought to be composed of non-transcribed nucleotide repetitions [1]. The possible roles that the satDNA could play within genomes are still controversial. However, enough studies point to their role in reproductive isolation and consequently with the appearance of new species [10,16] without a change in the chromosome number [17]. The way in which satDNA drives differentiation between species is still not well known but it is believed it acts in the chromosomal pairing in hybrids, playing the role of a “reproductive barrier” [11].

The classic procedure for isolating satDNA is the digestion of genomic DNA with restriction enzymes before separation by agarose gel electrophoresis. Occasionally, the use of restriction enzymes is unsuccessful due to the low copy number of the satDNA or because the repetition unit does not have the target sequence for the used enzyme [18]. Next-generation sequencing (NGS) techniques have opened a new way to characterize the repetitive DNA present in a genome. The repetitive fraction of a genome, defined as the “repeatome” [19] and in particular satDNA, today called “satellitome” [20] has always been underestimated [21] due to difficulties with assembling repetitive DNA regions. Novák et al. [22,23,24] developed powerful graph-based tools called RepeatExplorer and TAREAN that allow the analysis of the repeatome using direct reads from NGS, without the need to assemble the genome. In insects, the analysis of NGS data with bioinformatics tools like RepeatExplorer has been applied successfully, obtaining important results on the composition and evolution of the repetitive DNA in their genomes [17,20,25,26,27,28,29].

Beetles (order Coleoptera) are the largest and the most diverse group of animals, with more than 370,000 species, divided into four suborders: Archostemata, Myxophaga, Adephaga and Polyphaga [30]. Coccinelids belong to the Polyphaga suborder, known commonly as ladybird or ladybug beetles [31], comprising more than 6000 species distributed into 360 different genera and eight subfamilies, and found all over the world. Almost 90% of all the ladybird beetles are carnivorous and feed on aphids and other small insects, such as mealybugs, scale insects and sometimes mites [32,33,34,35,36]. Ladybirds are the most effective animals used in biological control [37]. In some ecological studies, the presence or absence of Coccinelids in olive cultures has been related to the state of conservation of the ecosystems [38] because they are very sensitive to changes in the pollution levels as well as pesticides [39]. *Hippodamia variegata*, Goeze 1777, also known as Adonis ladybird, the variegated ladybug and spotted amber ladybeetle, is one of the most commercialized ladybugs used for the biological control of aphid pests, including *Aphis gossypii* and *Myzus persicae*, which attack many economically important crops such as peach, cotton and tobacco [40,41]. This species is native in the Palearctic area but currently has also spread to other regions through introduction to control crop pests [42].

In spite of their ecological and economic importance, the data about repetitive DNA in Coccinelids are scarce. The analysis of satDNA has only been carried out in one Coccinellid species, *Chnootriba argus*, using classical methods such as restriction enzyme digestion or C0t-1 library construction [43,44]. The use of restriction enzymes allowed the isolation of a subtelomeric satDNA family located on the subtelomeric regions in all the chromosomes with the exception of the long arm of the X chromosome [43]. In the same species, but with the generation of a C0t-1 library, a satDNA family with an unusual short repeat unit with only 6 bp (TTAAAA) was isolated. This satDNA is also present in other Coccinellid species such as *Epilachna paenulata* but is not present in other species such as *Henosepilachna vigintioctomaculata*, *Henosepilachna septima* or *Diekeana admirabilis* [44].

In this study, the genome of *H. variegata* was sequenced and bioinformatics tools were applied in order to analyze its satellitome. Its economic importance and the small genome size of *H. variegata* (1C = 0.29 pg) in comparison with other Coleoptera species [45] makes it very suitable for the application of genomic analysis techniques. In addition, this study may be a first step that could help in the future to obtain the complete assembled genome of this important species.

## 2. Materials and Methods 

### 2.1. Sampling, Chromosome Preparation and DNA Extraction

*Hippodamia variegata* samples were provided by Biosur Insectarios (Murcia, Spain), a company focused on the production of predatory insects for biological pest control. Males were dissected and the testes were taken out for cytogenetic analysis. After dissection, the bodies were preserved in 100% ethanol at −20 °C until DNA extraction. Chromosome slides were prepared from male gonads obtained from adults [46]. Slides were stained with Giemsa, and analyzed with an Olympus (Hamburg, Germany) BX51 microscope equipped with an Olympus DP70 camera. C-banding was carried out following the protocol described by Sumner [47] including some modifications [48]. After C-banding, chromosomes were stained with DAPI (4’-6-diamino-2-fenil-indol).

Genomic DNA was isolated from adults using a commercial kit (NucleoSpin Tissue, Mini kit for DNA from cells and tissue, Macherey-Nagel Co., Düren, Germany) according to the instructions provided by the manufacturer. DNA concentration and purity were estimated by measuring the absorbance at 260 nm and 280 nm using a NanoDrop Lite spectrophotometer (Thermo Fisher Scientific, Waltham, MA, USA).

### 2.2. DNA Sequencing and Data Analysis

For genome sequencing, approximately 4 μg of genomic DNA were used. A library of 700 bp fragments were sequenced in the Illumina ^®^ Hiseq™ 2000 platform yielding about 3.2 Gb data of 101 bp pair-end reads. This Illumina library was deposited in the SRA database under Bioproject PRJNA644199.

A set of randomly selected 12 million pair-end reads (≈120 Mb) were used for the clustering and assembly analysis using RepeatExplorer, implemented within the Galaxy environment (www.repeatexplorer.org) [22,23]. We used default options, i.e., a minimum overlap of 55% and a similarity more than 90%. Clusters containing satDNAs were identified based on the graph topology with sphere or ring-like shapes. For each candidate cluster, we examined the contigs assembled by RepeatExplorer to search tandem repeated structures using the Dotmatcher tool (available on-line http://emboss.bioinformatics.nl/cgi-bin/emboss/dotmatcher/) and thus identify the monomer sequence for each satellite. Multiple-sequence alignments were performed using MUSCLE [49] to obtain the consensus sequences. All the obtained consensus sequences from potential satDNAs were used against Repbase using CENSOR (http://www.girinst.org/) [50]. The sequence data were also compared with the GenBank/NCBI DNA databases using the BLAST network service and the EMBL database [51]. We calculated the divergence and abundance for each satDNA using RepeatMasker [52] with the “-a” option and the RMBlast search engine. For this, we randomly selected a million reads and aligned against the total collection of satDNA dimers or monomer concatenations until approximately 200 bp in length was reached. We estimated the average divergence and generated a satellite landscape considering distances from the sequences by applying the Kimura 2-parameter model with perl script calcDivergenceFromAlign.pl and createRepeatLandscape.pl from the RepeatMasker suite.

### 2.3. PCR Amplification, Sequences Cloning and Cytogenetic Mapping

The consensus sequences of the most abundant satDNA families were used as a template to design a set of primers for PCR amplification (Table 1). PCR reactions were carried out in 20 μL reaction mixtures using 50 ng of genomic DNA, 0.5 mM dNTPs, 50 pmol of each primer and 1 U of *Taq* polymerase (Bioline, London, UK). The PCR program used was 2 min at 92 °C and 35 cycles with 20 s at 92 °C, 60 s at 50 °C, and 2 min at 72 °C, with a final extension of 5 min at 72 °C. After the amplification, the generated fragments were separated into 2% agarose gel electrophoresis. The expected bands were eluted from the gel using the E.Z.N.A.^®^ Gel Extraction kit (Omega, Norcross, GA, USA) and used for the ligation and transformation of competent *Escherichia coli* DH5α (Zymo Research, Orange, CA, USA) using the pGEMT-Easy vector (Promega, Southampton, UK). Recombinant plasmids were selected using LB/ampicillin/IPTG/X-Gal plates. The recombinant plasmids were purified using the E.Z.N.A.^®^ Plasmid Midi Kit (Omega, Norcross, GA, USA) and sequenced in both strands. Positive plasmids were labelled with biotin-16-dUTP using the Nick Translation Kit (Roche, Manheim, Germany), according to the company’s instructions. These labelled plasmids were used as probes (final concentration of 5 ng/ml in 50% formamide) to perform fluorescence in situ hybridizations (FISH) according to the procedure described in Palomeque et al. [48]. The fluorescent immunological detection was carried out using the avidin-FITC/anti-avidin-biotin system with three amplification rounds. Slides were mounted in Vectashield–DAPI (Vector Laboratories, Burlingame, CA, USA). DAPI in the antifade solution was used to counterstain the chromosomes. Images were taken with a BX51 Olympus^®^ fluorescence microscope (Olympus, Hamburg, Germany) equipped with a CCD camera (Olympus^®^ DP70) and processed using Adobe^®^ Photoshop^®^ software.

## 3. Results and Discussion

**Figure 1:**
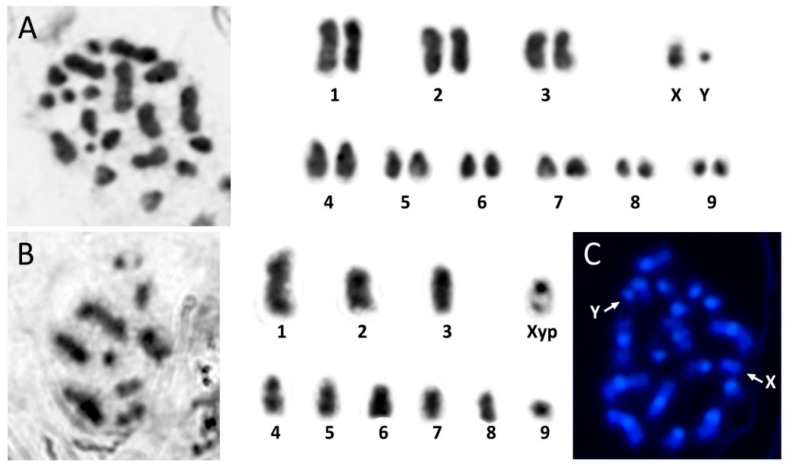
Mitotic (**A**) and meiotic (**B**) karyotype of *Hippodamia variegata*. (**C**) Mitotic metaphase stained with DAPI after C-banding showing pericentromeric heterochromatin in all chromosomes, including X and Y chromosomes.

*Hippodamia variegata* has a diploid chromosome number of 2n = 20, with a XY sexual chromosome system (Figure 1A). Sexual chromosomes show the parachute association Xyp in meiosis I (Figure 1B). Previously, Rozek and Holecová [53] reported the same chromosome number for populations from Central Europe. The karyotype of these specimens showed 7 pairs of subtelocentric and 2 pairs of metacentric autosomes, a metacentric X chromosome and the small Y chromosome that is dot-shaped. However, here we show that in the analyzed population, the karyotype presents three metacentric autosome pairs, the three biggest, and the metacentric X chromosome. All the remaining chromosomes seem to be subtelocentric or acrocentric, including the small Y chromosome (Figure 1A). These differences could be due to the existence of interpopulation polymorphisms. The small size of the chromosomes does not allow one to determinate the cause of this polymorphism, which probably could be provoked by a pericentromeric inversion. Chromosome polymorphisms due to variation in chromosome number or in the chromosome morphology were earlier described in other Coccinellidae species [54,55]. The chromosome number of *H. variegata* is similar to that found in other Coccinellidae species, where the most common chromosome numbers are 2n = 18 or 2n = 20 [56]. The Xyp system is also the most common among Coccinellidae although sexual determination systems such as XY, X0 and neo-XY systems have also been found [56].

Rozek and Holecová [53] reported that all the chromosomes of this species, after the C-banding, showed a big heterochromatic pericentromeric region with the exception of the Y chromosome that was completely euchromatic. However, we found that all chromosomes have relatively big pericentromeric heterochromatin and DAPI staining revealed that those heterochromatic regions are A+T rich (Figure 1C). The main component of the heterochromatin is satellite DNA, so in order to characterize the satDNA families presented in the *H. variegata*, we carried out NGS of its genome.

**Figure 2:**
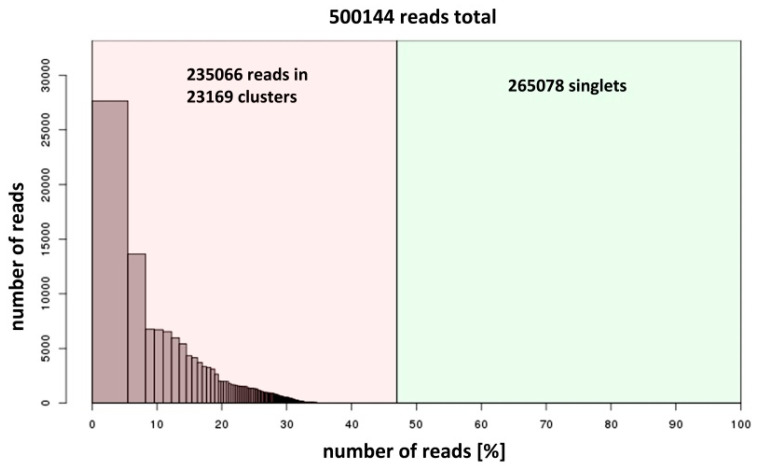
RepeatExplorer report showing the total amount of reads used as well as the number of clusters generated.

The sequencing data of the *H. variegata* genome produced 32,394,082 reads corresponding to more than 3 Gb. Sequencing data shows that the A+T genome content was 63.6%. This high A+T content is a common feature within the insect genomes, and can be over 70% in some species [57]. From the total reads, a subset of 12 million pair-end reads (≈1,200 Mb) was randomly selected to analyze in RepeatExplorer. The C-value of *H. variegata* is 0.29 pg [58]; therefore, the estimate haploid genome size of *H. variegata* is about 284 Mb. RepeatExplorer recommends using genome coverages of at least ~1% [22]. We selected 500,144 sequences in the pipeline, corresponding to about 5.8% of the genome of *H. variegata*. After RepeatExplorer clustering, 235,066 reads were grouped into 23,169 clusters, with 47% of the *H. variegata* genome composed of repeated sequences (Figure 2). All the remaining reads (265,078) were classified as singletons. From those clusters, 365 represented at least 0.001% of the genome and those that featured a star-like or circular graph topology, typically founded in the satDNA families, were deeply analyzed. After the computational analysis, we identified 29 satDNA families (Table 2, Supplementary Table S1) (Genbank accession no. MT613047-MT613075). The satDNA families were named according to Ruiz-Ruano et al. [20], including the species name abbreviation, a number in decreasing abundance and the length of the repeat sequence, starting from HvarSat01-277 (the most abundant) to HvarSat29-105 (the least abundant). Sequences with the insect telomeric repeat TTAGG were not found through this analysis. Nevertheless, the (TTAGG)_50_ repeat was included in RepeatMasker analysis showing that the abundance of telomeric repeats was 0.17% (Table 2). Consequently, the *H. variegata* satellitome is composed of at least 30 satDNA families, corresponding to 14.93% of the genome (Table 2).

Using classical isolation methodologies, only one or a few satDNA families have been characterized within a species [7]. However, the application of NGS technologies has eased the characterization of a large number of satDNA families. Among insects, for example, the grasshopper *Locusta migratoria*, it has been possible to find the existence of 62 families of satDNA [20]. Other analyzed Orthoptera species also present numerous families of satDNA, such as *Eneoptera surinamensis* with 45 families [28], 11 in *Gryllus assimilis* [29], 10 in *Ronderosia bergii* [59], 27 in *Eumigus monticola* [60] or the amazing 76 families found in *Pyrgomorpha conica* [27]. Orthoptera is the group of insects with the largest genome size, with genome sizes between 1.6 and 16 Gb [45]. In other insects, such as *Triatoma infestans* (Hemiptera), with a genome size between 1.5 and 1.9 Gb, NGS has revealed the existence of 42 satDNAs families [17]. One of the main causes of variation in the eukaryotic genome sizes is the amount of repetitive DNAs, including satDNA. However, the genome size may not be directly related to the number of satDNA families present in it. The Coleopteran *H. variegata* has a substantially smaller genome size (0.28 Gb) than the species discussed above and also has a large number of satDNA families. The number of satDNA families also does not seem to be related to the total amount of repetitive DNA in the genome. In *H. variegata*, the 30 satDNA families, altogether, comprise 14.93% of the genome. In the fish *Megaleporinus microcephalus*, 164 satDNA families have been isolated, which is, by far, the most satellite-rich species discovered to date. Nevertheless, the satDNA of *M. macrocephalus* only represented 13.47% (female) and 11.99% (male) of the genome [61]. Therefore, the existence of multiple satDNA families seems to be a common characteristic of eukaryotic genomes as they have also been found in plants and vertebrates [61,62,63]. 

**Figure 3:**
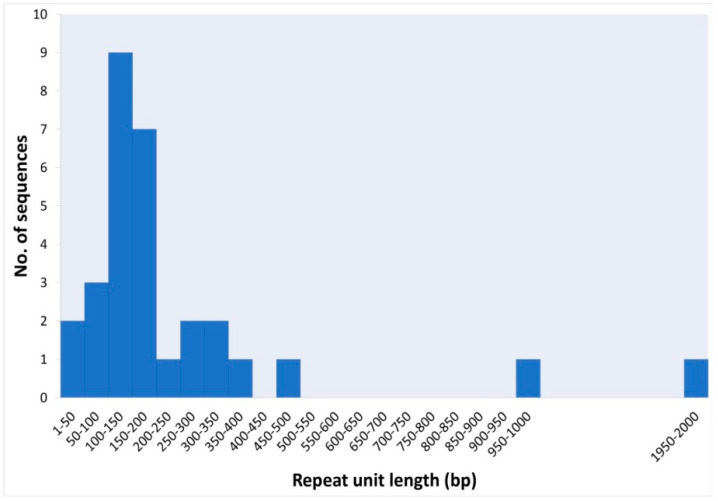
Size distribution of all the satDNAs characterized in *Hippodamia variegata*.

The *H. variegata* satellitome shows satDNA families that differ significantly in monomere size (Figure 3), from 5 bp (telomere) or 41 bp (HvarSat27-41), up to 2 kb (HvarSat07-2000). The most common repetitive unit size for *H. variegata* satDNAs is 100–200 bp. The family HvarSat07-2000, with a repeat unit of 2 kb is, to our knowledge, the largest repeat unit described so far in Coleoptera. Until now, the largest satDNA was the 1169-bp *Pst*I family isolated in the beetle *Misolampus goudoti* [6]. Satellites with the largest repeat units have also been described in other insect species, for example, in the ant *Monomorium subopacum*, with a repeat unit of 2.5 kb [64] or a satellite with a repeat unit of 1 kb found in the kissing bug *Triatoma infestans* [17], although most of the satDNA isolated in insects have repeat units over 500 bp [7].

The A+T content in the satDNA families of *H. variegata* ranges between 45.6 and 74.7% (Table 2). Traditionally it has been assumed that satellite DNA is rich in A+T, since most have an A+T content over 50% [7]. The A+T richness of the *H. variegata* genome is 63.6%. The number of satDNA families with A+T content above and below this value are equally distributed (Table 2). Therefore, there does not seem to be any tendency towards the enrichment of satellite DNA in A+T in relation to the complete genome. The HvarSat01-277 family is the main satDNA family in *H. variegata* and represents 9.37% of the genome (Table 2). The second most abundant family (HvarSat02-127) represents only 2% of the genome, with the remaining families below 1%. In fact, most satDNA families (21 out of 30) are below 0.1%. The existence of a main satDNA family, clearly the most abundant one, is a common characteristic of insect genomes [7]. However, there are species without a clear main satDNA family and different families of satDNA are located on the heterochromatic regions [20,28,29].

**Figure 4:**
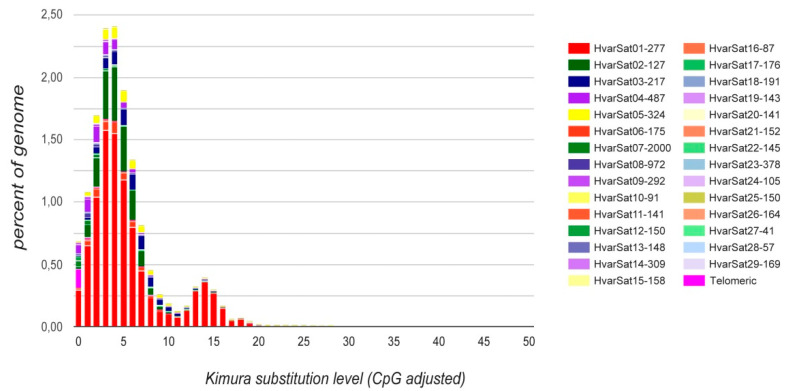
Satellitome landscape of the satDNA families in *Hippodamia variegata*.

Divergence of the *H. variegata* satellitome, estimated with RepeatMasker [52], is lower than in others insects in which the same methodology has been used [20,27,63]. The divergence among satDNA families ranges between 0.23% and 25.74%, with a median value being 5.75% (Table 2). The minimum values are shown by the telomeric repeat (0.23%) and the HvarSat07-2000 family (1.31%), whereas the highest value is shown by HvarSat16-87 (25.75%). Divergence of satDNA is inversely related to amplification and homogenization processes and directly related to mutation rate [62,65]. Consequently, low divergence in *H. variegata* satDNA families may reflect a tendency for homogenizations, which is also reflected in the satellitome landscape representing abundance (y-axis) and divergence (x-axis) for each satDNA with respect to each consensus sequence (Figure 4, Supplementary Figure S1). The main peak on the distribution shows that most satDNA sequences show divergence below 5%. That figure is shared for each satDNA family in separate repeat landscapes (Supplementary Figure S1). Interestingly, HvarSat01-277, the most abundant satDNA in *H. variegata*, shows two peaks corresponding with two types of repeat units differing in divergence (Supplementary Figure S3). The double peak pattern in a repeat landscape is not unusual, for example, in the fern *Vandenboschia speciosa* VspSat01-59, where telomeric repeats present two peaks on their repeat landscape [62].

Searches in Repbase using CENSOR (http://www.girinst.org/) failed to find sequences with significant similarity with the *H. variegata* satDNA families. Nucleotide sequences of the *H. variegata* satDNA families were also compared with the GenBank/NCBI DNA databases, obtaining only positive results for the HvarSat16-87 family. Sequences with similarity with the consensus sequence of HvarSat16-87 were found in two scaffolds obtained from the assembled genome of another Coccinellidae species, *Harmonia axyridis* [66]. These two scaffolds (accession no. AP018897, AP018898) contain five tandem arrays of repeated sequences (Supplementary Figure S2). From each tandem array we obtained monomers and generated a consensus sequence of 87 bp in length and 74.71% similarity with the consensus sequence of the HvarSat16-87 family. Interestingly, the HvarSat16-87 family is the most variable satDNA in the *H. variegata* genome (25.74% of divergence) and a similar variability was found for this repeat sequence in *Harmonia axyridis* (24.30% of divergence). These results are consistent with phylogenetic studies that show that *Harmonia* is the sister-genus of the genus *Hippodamia* [67,68].

**Figure 5:**
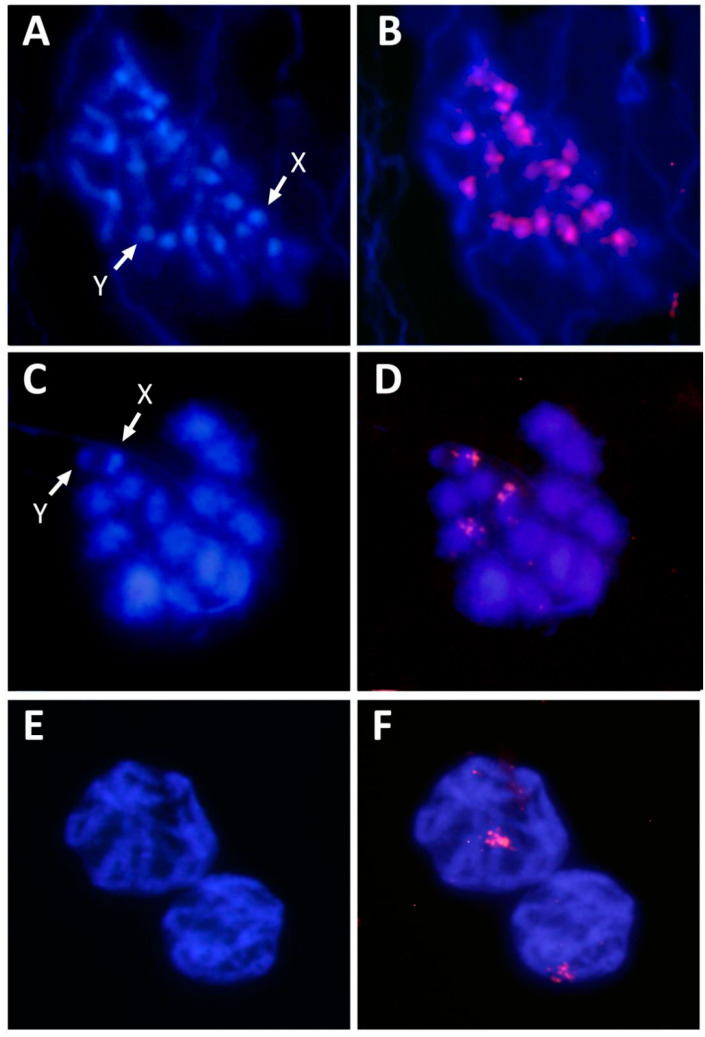
Chromosome location of the most abundant satDNAs of *Hippodamia variegata*. Mitotic metaphase stained with DAPI (**A**) and FISH with the HvarSat01-277 probe (**B**). Meiosis I stained with DAPI (**C**) and FISH with the HvarSat02-127 probe (**D**). Meiotic paquitenes stained with DAPI (**E**) and FISH with the HvarSat03-217 probe (**F**).

PCR amplifications for FISH probe generation were performed for the four most abundant satDNA families. Hybridization with the HvarSat01-277 probe shows a pericentromeric location on the chromosomes, coincident with the location of the heterochromatin revealed by C-banding and DAPI staining (Figure 5A,B). This result coincides with that observed in most satDNA families isolated from beetles, as they are often located in heterochromatic chromosomal regions rich in A+T. The most abundant satDNA is usually the main component of the heterochromatin located in the pericentromeric regions [4,14]. In addition, pericentromeric satDNA has an essential role in the formation and maintenance of kinetochorus, as well as in chromosome segregation and sister chromatid cohesion because it is involved in the stabilization of DNA-binding proteins [69,70,71].

Hybridization signals for the HvarSat02-127 family were located only on two small bivalents and the X chromosome (Figure 5C,D), whereas HvarSat03-217 signals were located on one bivalent only (Figure 5E,F). Due to their restricted location, these satDNAs could be very useful as chromosome markers, allowing one to distinguish those chromosomes pairs as well as the X chromosome from other chromosomes. No clear hybridization signals were obtained with the HvarSat04-487 satDNA probe. It is possible that this satDNA has a dispersed location in the *H. variegata* genome and therefore does not generate visible signs of hybridization. The existence of short arrays of satDNA along the chromosomes could result in the absence of hybridization signals or the presence of a bright background [20,27]. Traditionally, satDNA has been considered as DNA located on the heterochromatic regions, but euchromatin also possesses tandem repeats as microsatellites, minisatellites, and even satDNA [17,20,25,72].

In spite of their ecological and economic importance, the data about repetitive DNA in Coccinellidae are scarce as we indicated previously. *H. variegata* is the first Coccinellidae beetle whose satellitome has been studied by applying new sequencing technologies and bioinformatics tools. Recently, Louzada et al. [73] carried out a review of the important role that satellite DNA, along with other types of repetitive DNA, plays in the architecture and evolution of the genome. These authors highlight the importance of the application of the new technologies in the study of these sequences. 

Nowadays, knowing the composition and structure of genomes is a key not only for understanding their past evolution but also for discerning their future perspective. The *H. variagata* satellitome has shed some light about genome composition and structure on this species. Furthermore, it would be helpful for mapping scaffolds to chromosomes in a future genome assembly project, leading to a complete and well-annotated genome.

## Figures and Tables

**Table 1:** Designed primers for PCR amplification of the four main satellite DNA (satDNA) families of *Hippodamia variegata*.

SatDNA Family	Primer Name	Primer Sequence
HvarSat01-277	Hvar-CL1-F	5′ ACTCTCTATCCCTACCCG
	Hvar-CL1-R	5′ AAATCAGTTGAGCCTGAG
HvarSat02-127	Hvar-CL3-F	5′ AAAAATCGAGAGTTTTCG
	Hvar-CL3-R	5′ TTCATCTCATTCTGACGC
HvarSat03-217	Hvar-CL13-F	5′ CTGGCAAATGCAAGACTTC
	Hvar-CL13-R	5′ TACGATGTATTCACGACG
HvarSat04-487	Hvar-CL12-F	5′ GAAGATCTGATCCCACTG
	Hvar-CL12-R	5′ TCAGAGGCAATCTAGGAG

**Table 2:** Data of the satDNA families found in *Hippodamia variegata*: genome abundance (%), length of the repeat unit, A+T content and divergence (%).

Name	Genome Proportion	Repeat Unit Length (bp)	A+T Percentage	Kimura Divergence (%)
HvarSat01-277	9.37%	277	65.3%	5.71%
HvarSat02-127	2.04%	127	65.4%	4.46%
HvarSat03-217	0.92%	217	59.9%	6.67%
HvarSat04-487	0.62%	487	64.1%	3.25%
HvarSat05-324	0.56%	324	59.9%	5.78%
HvarSat06-175	0.49%	175	65.1%	4.91%
HvarSat07-2000	0.13%	2000	68.2%	1.31%
HvarSat08-972	0.10%	972	66.8%	2.38%
HvarSat09-292	0.09%	292	59.9%	3.73%
HvarSat10-91	0.07%	91	63.8%	9.35%
HvarSat11-141	0.07%	141	63.9%	4.89%
HvarSat12-150	0.05%	150	52.7%	1.19%
HvarSat13-148	0.04%	148	58.1%	6.68%
HvarSat14-309	0.03%	309	70.3%	21.34%
HvarSat15-158	0.03%	158	63.2%	8.45%
HvarSat16-87	0.03%	87	74.7%	25.74%
HvarSat17-176	0.024%	176	65.2%	10.22%
HvarSat18-191	0.020%	191	49.2%	4.44%
HvarSat19-143	0.018%	143	64.4%	5.79%
HvarSat20-141	0.016%	141	60.3%	7.51%
HvarSat21-152	0.015%	152	62.5%	8.63%
HvarSat22-145	0.011%	145	63.5%	9.27%
HvarSat23-378	0.011%	378	60.4%	2.28%
HvarSat24-105	0.008%	105	53.3%	2.98%
HvarSat25-150	0.007%	150	69.3%	8.2%
HvarSat26-164	0.006%	164	66.4%	5.11%
HvarSat27-41	0.004%	41	68.3%	9.04%
HvarSat28-57	0.003%	57	45.6%	6.86%
HvarSat29-169	0.003%	169	69.2%	5.5%
Telomeric	0.17%	5	60.0%	0.23%
**Total**	**14.93%**			
Mean		265.73	62.63%	6.73%
SD		372.02	6.27%	5.30%
Median		155.00	63.85%	5.75%

## Supplementary Materials

The following are available online at https://www.mdpi.com/2073-4425/11/7/783/s1, Figure S1: Separate repeat landscape of each satDNA family in Hippodamia variegata satellitome; Figure S2: (A) Dotplot of an internal region of one of the scaffold of Harmonia axyridis with the presence of a tandem array of repeat sequences with similarity with the HvarSat16-87 satDNA family of Hippodamia variegata. (B) Alignment and consensus sequence of repeat sequences found in two scaffolds of Harmonia axyridis (accession number AP018897 and AP018898). (C) Alignment of HvarSat16-87 and the consensus sequence of the repeat sequences found in Harmonia axyridis; Figure S3: (A) Repeat landscape of the Hvar-Sat01-277 satellite DNA showing the existence of two types of repeats. (B) Alignment of the consensus sequence of the Hvar-Sat01-277 with the most abundant repeats (type I) and with the less abundant (type II); Table S1: Consensus sequence and accession numbers of the satDNA families found in *Hippodamia variegata*.
