# Supplementary material for: Satellitome Analysis in the Ladybird Beetle Hippodamia variegata (Coleoptera, Coccinellidae)
Source: Genes (Basel). 2020 Jul 13;11(7):783. doi: 10.3390/genes11070783 (PMC7397073; doi:10.3390/genes11070783)
Supplement: Supplementary file 1 [file genes-11-00783-s001.pdf]

Supplementary Material

Supplementary Figure S1. Separate repeat landscape of each satDNA family in *Hippodamia variegata* satellitome.

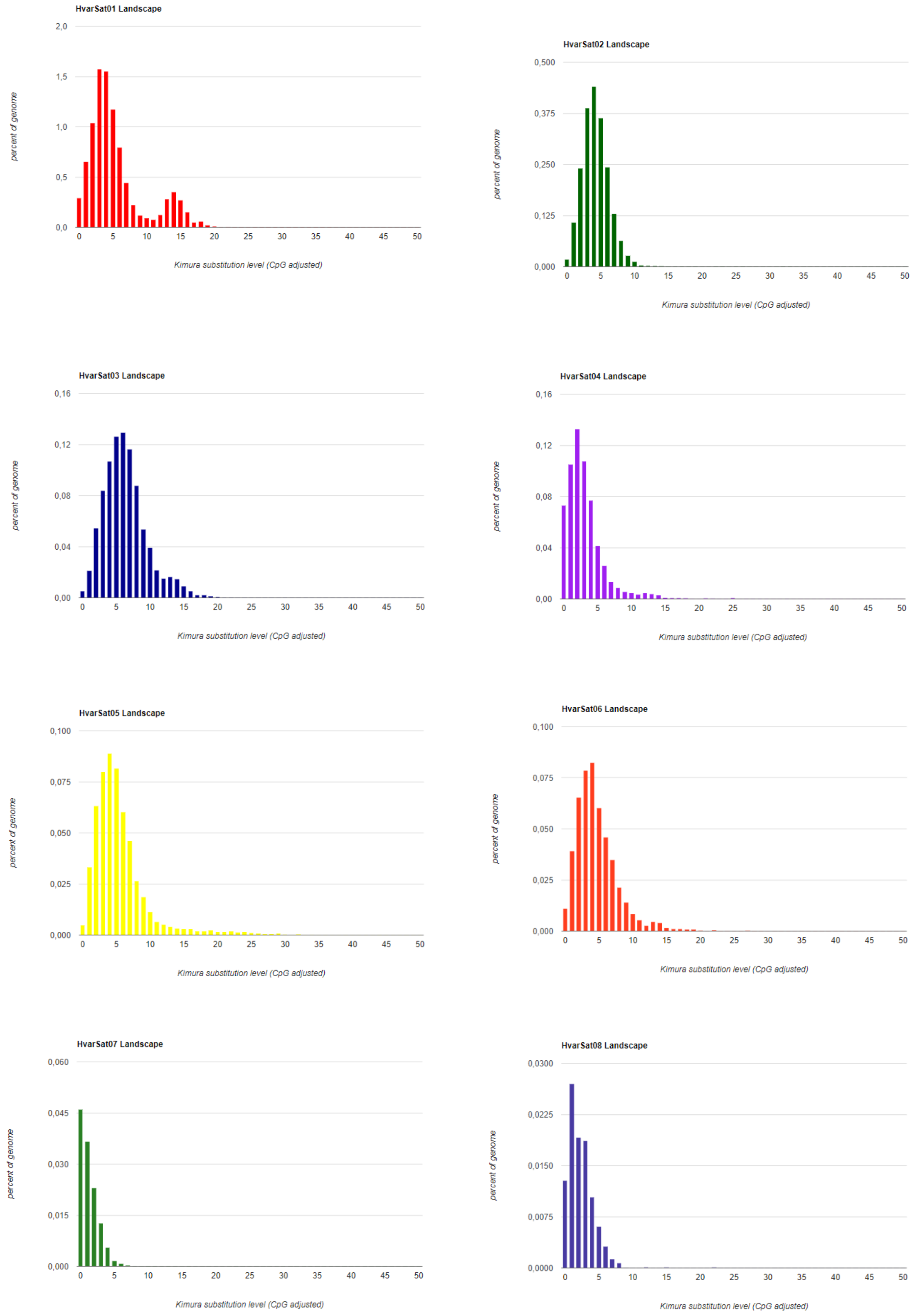

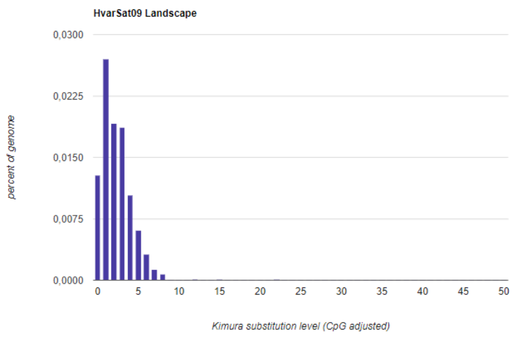
HvarSat09 Landscape
0,0300
0,0225
0,0150
0,0075
0,0000
percent of genome
0 5 10 15 20 25 30 35 40 45 50
Kimura substitution level (CpG adjusted)

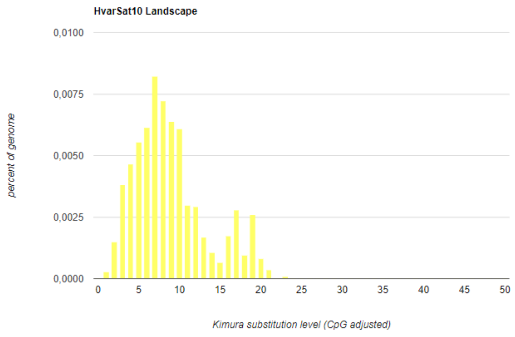
HvarSat10 Landscape
0,0100
0,0075
0,0050
0,0025
0,0000
percent of genome
0 5 10 15 20 25 30 35 40 45 50
Kimura substitution level (CpG adjusted)

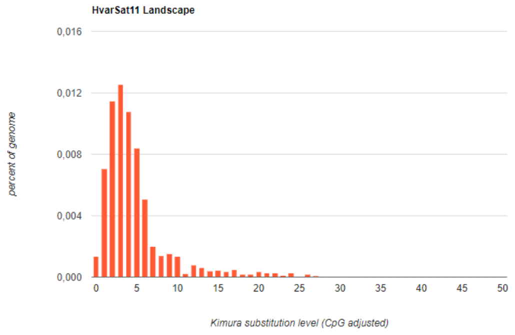
HvarSat11 Landscape
0,016
0,012
0,008
0,004
0,000
percent of genome
0 5 10 15 20 25 30 35 40 45 50
Kimura substitution level (CpG adjusted)

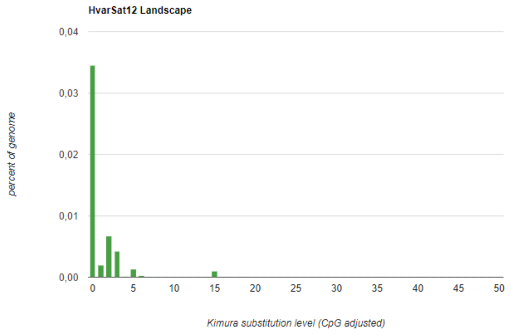
HvarSat12 Landscape
0,04
0,03
0,02
0,01
0,00
percent of genome
0 5 10 15 20 25 30 35 40 45 50
Kimura substitution level (CpG adjusted)

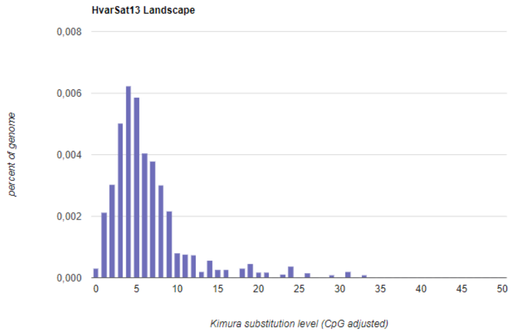
HvarSat13 Landscape
0,008
0,006
0,004
0,002
0,000
percent of genome
0 5 10 15 20 25 30 35 40 45 50
Kimura substitution level (CpG adjusted)

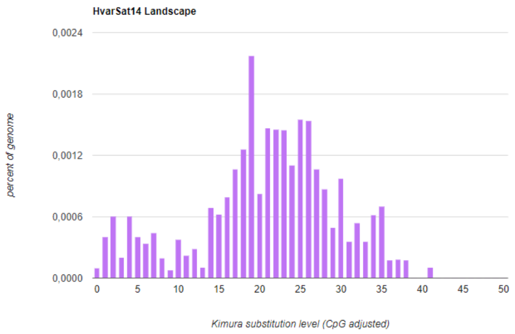
HvarSat14 Landscape
0,0024
0,0018
0,0012
0,0006
0,0000
percent of genome
0 5 10 15 20 25 30 35 40 45 50
Kimura substitution level (CpG adjusted)

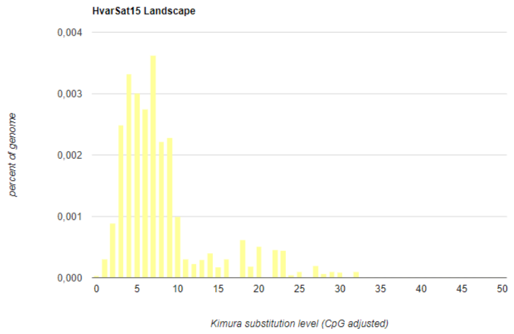
HvarSat15 Landscape
0,004
0,003
0,002
0,001
0,000
percent of genome
0 5 10 15 20 25 30 35 40 45 50
Kimura substitution level (CpG adjusted)

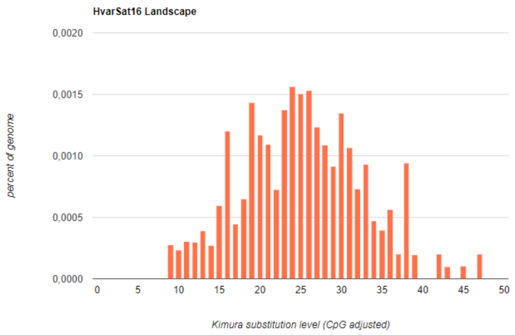
HvarSat16 Landscape
0,0020
0,0015
0,0010
0,0005
0,0000
percent of genome
0 5 10 15 20 25 30 35 40 45 50
Kimura substitution level (CpG adjusted)

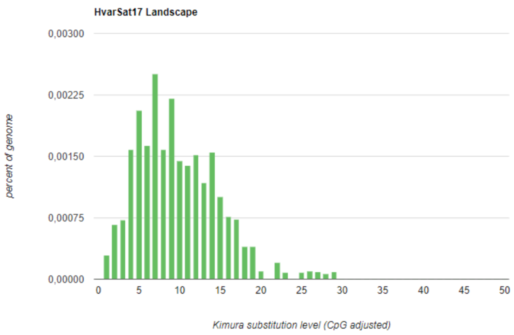
HvarSat17 Landscape
0,00300
0,00225
0,00150
0,00075
0,00000
percent of genome
0 5 10 15 20 25 30 35 40 45 50
Kimura substitution level (CpG adjusted)

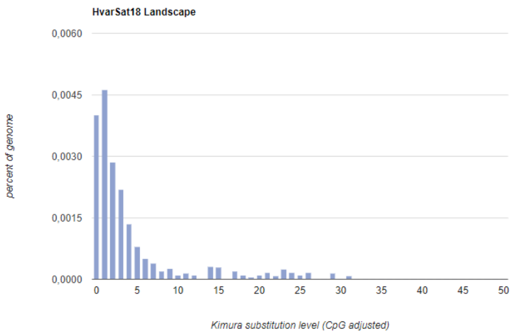
HvarSat18 Landscape
0,0060
0,0045
0,0030
0,0015
0,0000
percent of genome
0 5 10 15 20 25 30 35 40 45 50
Kimura substitution level (CpG adjusted)

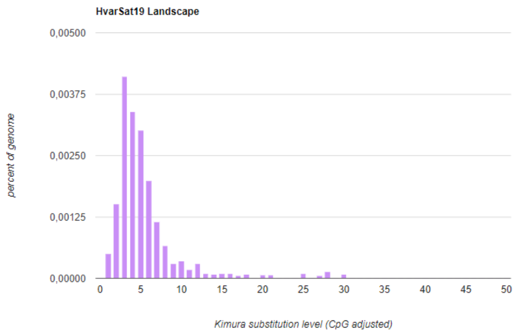
HvarSat19 Landscape
0,00500
0,00375
0,00250
0,00125
0,00000
percent of genome
0 5 10 15 20 25 30 35 40 45 50
Kimura substitution level (CpG adjusted)

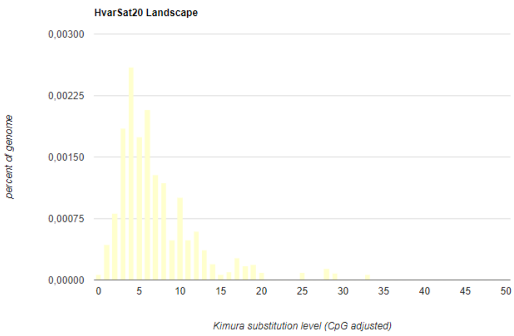
HvarSat20 Landscape
0,00300
0,00225
0,00150
0,00075
0,00000
percent of genome
0 5 10 15 20 25 30 35 40 45 50
Kimura substitution level (CpG adjusted)

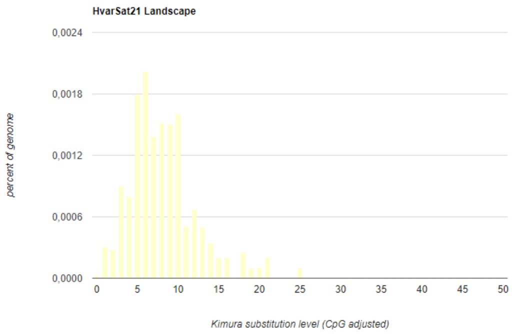
HvarSat21 Landscape
0,0024
0,0018
0,0012
0,0006
0,0000
percent of genome
0 5 10 15 20 25 30 35 40 45 50
Kimura substitution level (CpG adjusted)

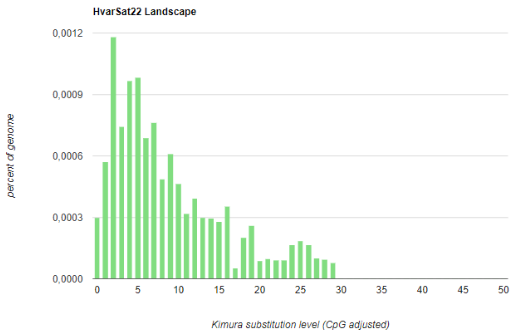
HvarSat22 Landscape
0,0012
0,0009
0,0006
0,0003
0,0000
percent of genome
0 5 10 15 20 25 30 35 40 45 50
Kimura substitution level (CpG adjusted)

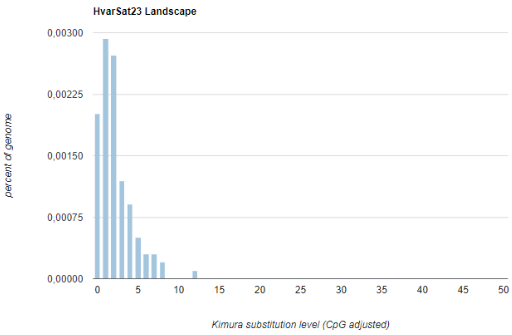
HvarSat23 Landscape
0,00300
0,00225
0,00150
0,00075
0,00000
percent of genome
0 5 10 15 20 25 30 35 40 45 50
Kimura substitution level (CpG adjusted)

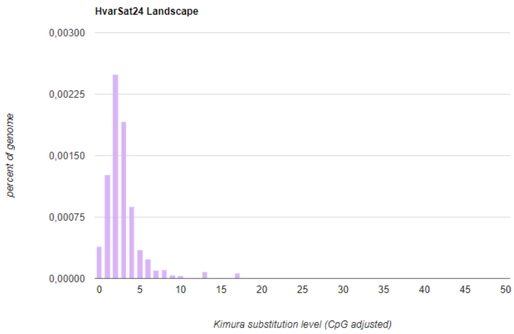
HvarSat24 Landscape
0,00300
0,00225
0,00150
0,00075
0,00000
percent of genome
0 5 10 15 20 25 30 35 40 45 50
Kimura substitution level (CpG adjusted)

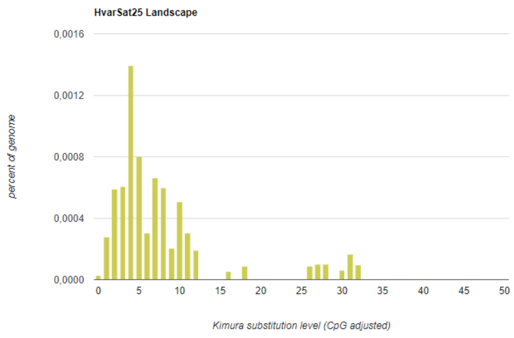
HvarSat25 Landscape
percent of genome
Kimura substitution level (CpG adjusted)

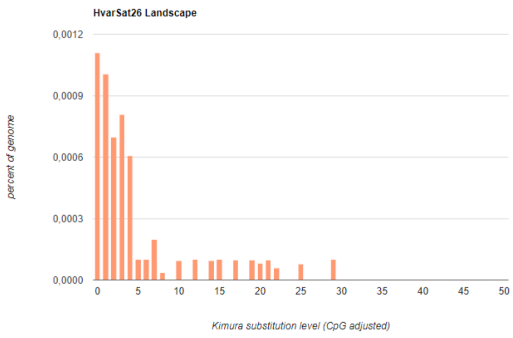
HvarSat26 Landscape
percent of genome
Kimura substitution level (CpG adjusted)

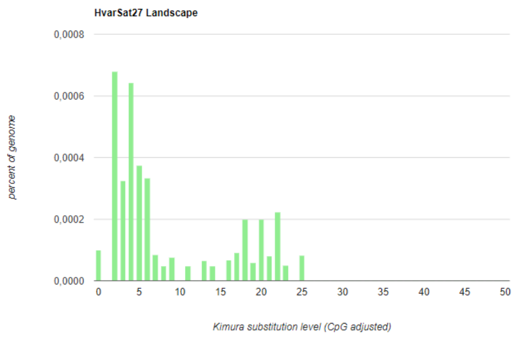
HvarSat27 Landscape
percent of genome
Kimura substitution level (CpG adjusted)

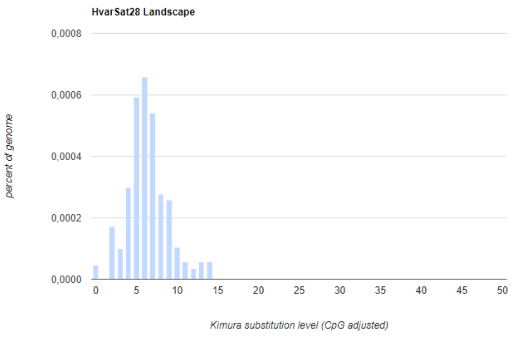
HvarSat28 Landscape
percent of genome
Kimura substitution level (CpG adjusted)

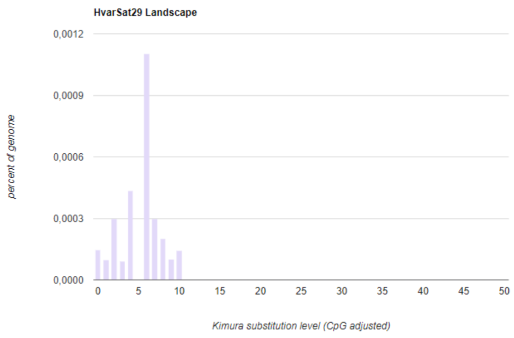
HvarSat29 Landscape
percent of genome
Kimura substitution level (CpG adjusted)

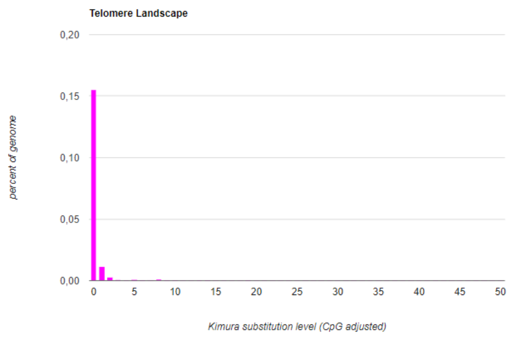
Telomere Landscape
percent of genome
Kimura substitution level (CpG adjusted)

Supplementary Figure S2. (A) Dotplot of an internal region of one of the scaffold of *Harmonia axyridis* with the presence of a tandem array of repeat sequences with similarity with the HvarSat16-87 satDNA family of *H. variegata*. (B) Alignment and consensus sequence of repeat sequences found in two scaffolds of *Harmonia axyridis* (accession number AP018897 and AP018898). (C) Alignment of HvarSat16-87 and the consensus sequence of the repeat sequences found in *Harmonia axyridis*.

A

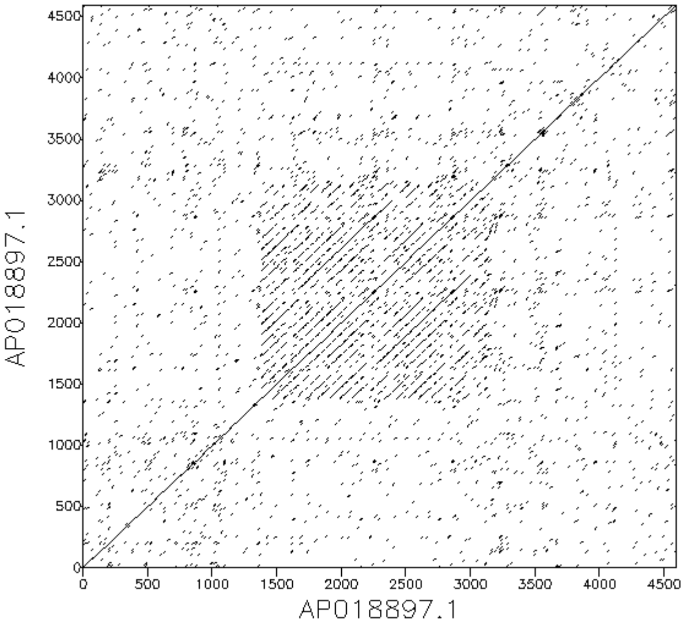

B

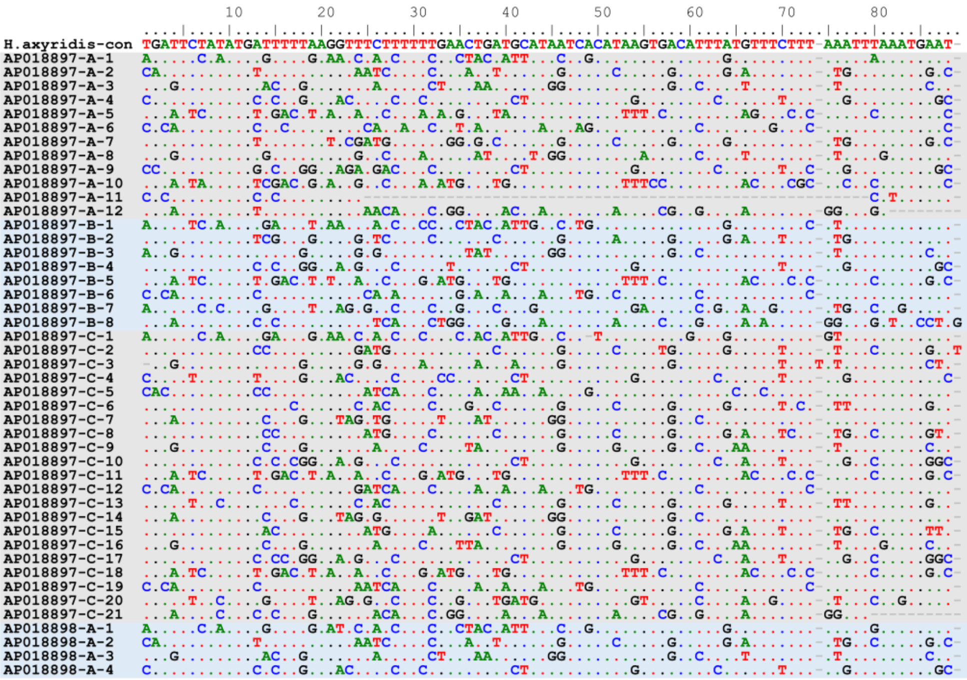

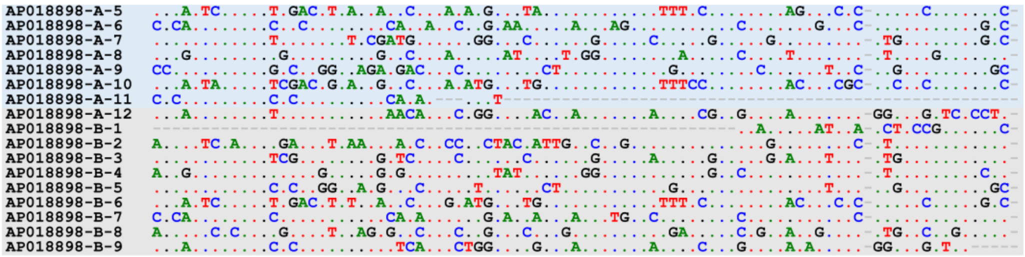
AP018898-A-5
AP018898-A-6
AP018898-A-7
AP018898-A-8
AP018898-A-9
AP018898-A-10
AP018898-A-11
AP018898-A-12
AP018898-B-1
AP018898-B-2
AP018898-B-3
AP018898-B-4
AP018898-B-5
AP018898-B-6
AP018898-B-7
AP018898-B-8
AP018898-B-9

C

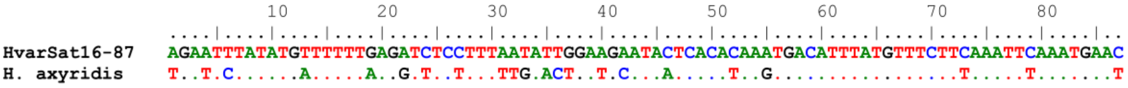
HvarSat16-87
H. axyridis

Supplementary Figure S3. (A) Repeat landscape of the Hvar-Sat01-277 satellite DNA showing the existence of two types of repeats. (B) Alignment of the consensus sequence of the Hvar-Sat01-277 with the most abundant repeats (type I) and with the less abundant (type II).

A

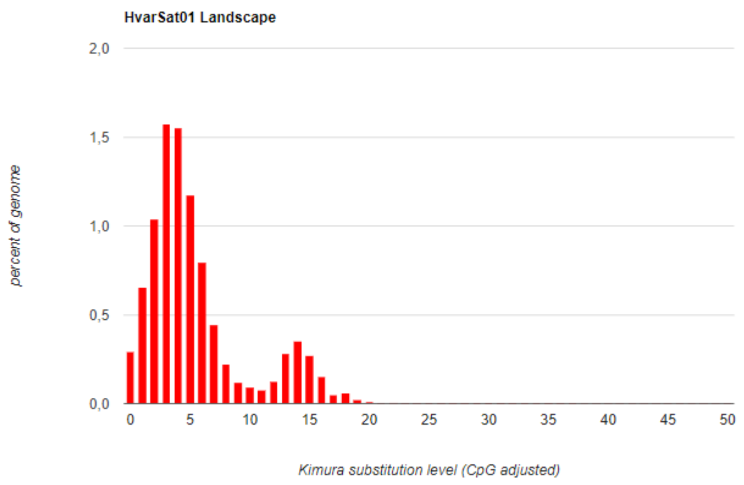

B

```
                        10        20        30        40        50        60        70        80        90       100
                ....|....|....|....|....|....|....|....|....|....|....|....|....|....|....|....|....|....|....|....|
Hvar-Sat01-277  TCAAATCTCAACTCTCTATCCCTACCCGAAGTCGAGATAATCATGTCAATCATGAATGCACGAAATCATCAAGAACGAAGCATTTTATGGTTTCAGTTCT
Type I          .............T..........G...........................................................................
Type II         ....................T.............................................................................T.

                       110       120       130       140       150       160       170       180       190       200
                ....|....|....|....|....|....|....|....|....|....|....|....|....|....|....|....|....|....|....|....|
Hvar-Sat01-277  TCGATTCACCCTTATTGACCAATAACATAGTGTTATAAATGCATTTGAATGTGTTTAACAGATTCAAAATCGGAGAAAAGAGTTCGTAATAGGAAATGTC
Type I          ................................................................................T......C............
Type II         ......T..A............................................................GA............................

                       210       220       230       240       250       260       270
                ....|....|....|....|....|....|....|....|....|....|....|....|....|....|....|..
Hvar-Sat01-277  AAGTTTAACAAGATGGAAGTACCGAAATGACTTGAAATTTCATAATTCTCAGGCTCAACTGATTTCTACTCGATTTA
Type I          ......G.........G....TA.GT..........................A.......................G
Type II         .....C............AC...........C................G..........................C.
```

Supplementary Table S1. Consensus sequence and accession numbers of the satDNA families found in *Hippodamia variegata*.

**Accession number**	**Consensus sequences**
MT613047	>HvarSat01-277<br>CTCGATTTATCAAATCTCAACTCTCTATCCCTACCCGAAGTCGAGATAATCA<br>TGTCAATCATGAATGCACGAAATCATCAAGAACGAAGCATTTTATGGTTTCA<br>GTTCTTCGATTCACCCTTATTGACCAATAACATAGTGTTATAAATGCATTTGA<br>ATGTGTTTAACAGATTCAAAATCGGAGAAAAGTGTTCGTCATAGGAAATGT<br>CAAGTTTGACAAGATGGGAGTATAGGAATGACTTGAAATTTCATAATTCTCA<br>GACTCAACTGATTTCTA
MT613048	>HvarSat02-127<br>TTTTTGTTATTTTCGAAAACTCTCGATTTTTCTGATTACAAAACAGTAACATT<br>CATCTCATTCTGACGCTGACGAAACGAGATATTCAGGTTTGAAGGTTCTTGA<br>ATTTCCACTTCGGATTCTGGTT
MT613049	>HvarSat03-217<br>TTATAAAAGGAGCCACTCGATCGATAGATGATCATTTCAAATCGGAATAGC<br>GTCGTGAATACATCGTAGGTAGAGTTGAAAATTTAATAAACTTTCAATAAA<br>ATTTTCAGGTGATAGACCAGTCCTGCAAATGCAAGACTTCATTGTGTAGTTG<br>CGTCACTCTACTCGAAAATACTACTCGGTCGCGTTGTTACGTCACAGTTACG<br>TCGGGGGTAGC
MT613050	>HvarSat04-487<br>TTATGGAGGTCATAAAACCGGTCGAGGTGCTCCAGAAAAAAATACCAACTT<br>GAATTATTTTACCCATATTGTGGTCGGTAGAGCGTCTCAAAGTCAACTCGAA<br>AATGAAAAATGGAAAAACAACTTTATTTTTTTTCGCGAAAACTAGGACAG<br>ATAATTCCGGGGTTGTAATTGAAATTGTATATTACTCGATTGCTTCATCTGAT<br>GAAGGCAAAATAATTGCACATTTCTCGATTTTAAAATTTTTCACCTTCAAAA<br>TGAAAATTTTCAAATTTCAAACTTGTATATTTTTGGCGCCATCGCGGCAGATC<br>GATGCGAGGTTTTCGGAAAGGTCATCAGTGGGATCAGATCTTCAATCCAAG<br>CACAAAAAATCGCAAAAAAAAATTTTGAAATTTTGACCGAAAAAATCGTAA<br>GGGGTTGACCCCCCCTAAAACGCTATATTTTCCATTCTCAGAGGCAATCTAG<br>GAGAGCTATCGTAATTGTTTG
MT613051	>HvarSat05-324<br>GATCGGATTCAAAACACAGGAGCTAGAGCAGATTATAGATTTTCCCTACGC<br>GAACCACATCGTTTCAGGTAAATTCCTGATTTTCAAGTCTGGTTGATATTTTA<br>GGGTGAAAATGATATTATTTTTGAAATCTGCGACCCCGAATTGACCTGAAAC<br>GATACCCCACATCCCCCCAGAAGGATTAGAAATTTTTTCGAAAATCCCTATC<br>TTACATAGGTCGGTGACCCAAAAAGTGCGTTTAAAAATTTTTTCGGAATAT<br>CGGGCTGAAAATCAGAACATAGGTAGAAGAGGCCGTGGTGTTTCATATGCG<br>ACTAGATCAGCTC
MT613052	>HvarSat06-175<br>ATATTCTGTAAAATATTCATCCCGATAGGCCCCCGTCTTCATAGAAAAAATC<br>GAGAAAAACAGTAAATTTTTCACTCAAAATTCATTAAAATTCATTAAAAATT<br>GAAAAAAAAGGCCCTTACACCGTATGTAGGGGCATTATTGTACCTTCAAAA<br>GATGGAAGACACCGAGGCGC
MT613053	>HvarSat07-2000<br>CAGAATATAAATAAATTCACAGTCAAGTTAATACAGAAAAACACATATTTA<br>CATATCTACAATAATTACAAATGATCGATAGAAAAAAAAAGCATAATTCTA<br>ACCCATCAATATTTTACATGGGGCGTCAGAAATTGAATAATGTGAAAAATT

	AATTCAAAATATGAAAATTGATGAATAATCAGTCAGGTATATAACAAAAAG<br>AAACATATTCATATATCTACAAAAACTACGAACGATCAATAGGAAATAAAA<br>CATATCGTGAATTTCTCAACCTATCGCCATTTTTACGTTCAGTTTTCAATACG<br>AACAAGACCAATTAAGCCTAGGCATGGAATTCACATGATTCCGCCTCATAT<br>GCTTGATGGTATTTTTCAATAAAATTATCATTCTCAATCTGTCACATTCGGGT<br>ATTCCTGACTTTTTTTTGTGTTTTCGTGTTGGTATTCTGAGCACTTTCAATTTTT<br>CTAGAAAGCGTTTTTCCAGTTCATTTGGGCTTATATCATAATCACTGATGTAT<br>AATTCAAATTTATCACTATGAAGCAATGATTCATTGCCTTGATTTTCATAGGA<br>TTCGTTTTCGTCACTGATTTTCAACAATTTCAAAATTTTCGAACACTCAACTT<br>GTTCGTTTACTCTGTTATTTATTTCACTGCATATCACAGGATTTGTCATTGTTT<br>TTTCACAAGAATCGTTGAAGTCTATTTTTCTGGCAGTGCTGGATCTGGGAAA<br>TTTAGGGGCTTGAGAATTCGAGCTTTTCGAAGGCTTTTTCACCCAGTGGCTTG<br>CGGGTGCGGCATTTTTCAGTTTTTGCTGAGCCTCGTAAAAATTCAAAACGTG<br>ACGCTGTATTTTGGTATGAACGGTTTGCTTCGTTTTCTGCTGTACCCTCAATCT<br>GTCTGGAAATAATTTTCCTTTTTCTCTCGTCATATCATTTCTACTCAATATAGA<br>AATTATGTCAGTGCATGAAGTTTCACCTATATTTTCAGATGAATTGTTCGAAT<br>CAACCATTTTGGTATTCATTGTTCGTTGCATACTCCATCTGCCCACATAGACT<br>TGTATTGGGTCTTTCCTCAAAATTTCATGGGATTCACTTTCTTTGTAGGCAGA<br>AGCATCGTTCCCTGACCATTGATCAATTTCTCGTGTTGTTGAAGTATTCATTG<br>TGATACCGTCCAATCTGAATTAGAATTAAACTTAATATTGATATCTTAGTTTC<br>AACGAACACTGAGGAAAAATTATTTCAATATTTAAAAAAGGATTGAAATGG<br>AAAAGATATTTGAAAATTATATTTCACAACAATTATGAGATATTCAACATAA<br>AAATGGATGATTGGATTTCAAATCCACGAAATGGAAATAAATTGACAAAAA<br>GACGATTTTCAAATTTTATATCGATGATAATTGAACTGTTCGAAAACTCAAT<br>TCATGTATCATCAAAAATATTTTTGATATATGATGAAAATCAAGAGATGAAA<br>ATAAAACAAATATCATAAACGAAAATTGTGCAATTTGAACTCACCCACGTA<br>TATTTCAACCACACTTATTCAAAACTACAACTTTTCGTTCCGTTCACTTAGAA<br>ATATAGATAGCACTAAATGTGACACCTTATAGCGCTCTCTATTTAAGTACTTT<br>CGCTTTCAGGATTCTCGGAACTATTATGACAAAGGGTAGTTCTGAGAAGGTA<br>CATTATCTCGGTTCCTAATCTTTGTTTGGTATCTAATCGGTATCATGTACTCGG<br>TAATTTCGAAATATCTACTACAAAGGGTAGTTCTGAGAAGGTAAATTATCTC<br>GGTTCCTAATCTTTGTTAGGTATCTAATCGGTATCATGTACTCAGTAATTTTG<br>AAATTTTCATCACAAAGGATAATTCTGGAGAGATATTTTATCTTTCACAGAA<br>ATATCGGTACCAGAACTTTGTTGGGCATCCGATTATCTTGGTATCAGTAAAA<br>ACAATCTTTATATTAAGTAATTATGTAAAAGTATTATATCTGACTGGGAAAT<br>CTCAGTAC
MT613054	>HvarSat08-972<br>TGAATTTACTGAGTGACGGCATAATTATATGGTCGATTATAGAATATAAGAT<br>TTGTTTTCAAATCTGTATGATGAACTCAAGAATGGTTCCAGAGGGAACCATT<br>CTTGAGGTATTAATTTACTGATCAAATTTTTTTCTTCAGAAAATGAGACAAAT<br>TCTAGGAAATACTTTTGTTTCGCCTAGGATAAACAACTTACTGCTCACAATA<br>GATATAGCTTGATGATCAAGTGAAAGGACACCGACATTGAGAACTGAATTC<br>TAACTACCTGTTAATGGAATCTGGATGAATGTTATCAATATCCGACAATCAA<br>CCTTTCAGGCAGGAAGTGACTTTGTTTTCAACACTGTATGATAAACAAACCT<br>TTGGAACCATTCTCAAGGTGTACATTAAGTGATTTGAGATAAGAAACCGTCT<br>CTAACAAAAGAGGAGATAATCATTCTAGGAAAATAATTATATTCGGAATGA<br>AAACATCTCTCTAATAAGCACAATCTGAAACAAGAAGTAGAAGTCAGTGCG<br>GTTTGGTTGGTAGAATCATACGTTGTACATCAGTTGCGATGATGAAAGTTCA<br>AGCAGGTGAGGACAAACATAACATTATGACTACTTCCATGAGAAAGGAATT<br>AATTTATGAAATCTTCGAGCAGGGTAATATTGTGAGTGAGAATAATTTTCAT<br>TCCCAATCTCTTTATTTCGATTCATCGAATCAATTTCTGGTTTTCGGCGGAAA

	TATCAGAAATTTATCTATCAAAGATCCAGAACAAGTTATCTATCGACCCACA<br>ATATACATTTGTGACTTGGAGAAATTAATCAGAAATTCATGAGTCTTGAGTT<br>TTTCATTCGACTATCTTGCGAAATTTTCGGTTTCATATTGACAAAATATTCAA<br>ATTTCCGTATGTTAAATCTACTGAATGACGGCATAATTATATCGTTGATTATA<br>GAATACAAGATTTGTTTTCAAATCTGTATGATAAAC
MT613055	>HvarSat09-292<br>AATTCGGGGTCGTAGATTCATAAAAACTGGTTATTTGGGCTCTAGAGGGTGA<br>ACCAAGTGGATTTTTCTCTAAAAACATGGGTTCCAAGGACTCCTTGACACTT<br>TGGAGCGGAAAGGACCTGATTTTCTGAATCTACGACCCAGATTTCCTATGAA<br>ATGACACCCAACATCGCCTATGTATGATGAAAAATTTCGACTCGGTTCCAAC<br>CTTCGTAAGAGAAAAATCAAATTTTTGTGAAAAACGTCAAAACTACTCATC<br>GTAGGTGGGTCATGTTGGGTATCAATTCATACT
MT613056	>HvarSat10-91<br>TTCATTGTTGGCACAGGAAGTGAGGAATGCAAAAATTTCTACTGGCTCTACT<br>CAACTGCCTAGCTCAAATATCCACCATTATTTTTATTAA
MT613057	>HvarSat11-141<br>CAACCATATTTCGTATGTGGGATCCTTATATCAATGTGATTAACTGATAAAA<br>AAATCATCGACTCGCAAAGGGTTTCAGGGGGTGAAAAATCAACCCTTAAAC<br>TGCAAAATTCTATATCTCGGAAACAATTCGGAGTATCA
MT613058	>HvarSat12-150<br>ACTAGTATGGCCCGGGGGATCCTACGTTCCAAATGCAGCGAGCTCGTATAA<br>CCCTTTAAGAGTTGCTCTTTTTGTTTGGTAAGTTGCAAATCGAAGTTTTAGAT<br>TGAGTTCTACGTCGAGCGGCCGCGATATCCTGCAGATGCATCCAGT
MT613059	>HvarSat13-148<br>TCTATTTTCAAAAACTTCTCCTGAGTGCGTAAAAAGTCGAATTTCTGAAATTT<br>TTTTTCGGTGAATATATCCCTCTGAACGCCCTCTAGAAGATCCACATATCCA<br>CATATTTTGTCGGGCAAAACTTCACCCCCCACCACACCCCTCC
MT613060	>HvarSat14-309<br>GGATGAAAAGTTGACAACTGTCGATATATTCAGAGTTATGAGGTATTCCCTT<br>CAATATAATTCAGAAATGTTTTGATTCACTTGGATGTTTTTTATGTCAATTTC<br>AATATCTATCAATTCAAGTGAGTTAAAATATTCGTTACATCCGAAAAGTTGT<br>TTTCGAATAGAAATAAGACGATTTCGTGTATTCACAATATTTATTTCTGAAA<br>AGAAAGACTTCATAACTCTCGGTTTTAGAATGAAACTCATTTCGATTGCGAT<br>GCATATTGAAAATCGGCTCGAAATGAGATTTCCAAAACAAAATATTCA
MT613061	>HvarSat15-158<br>TAATTAACACCAAAAACCTAATCAAGATATGCGACAACGTCTTTTTTTGATT<br>TTAGAGAACTTTGAATGTTCGAAGAACAGAATCTTTCGAAGTCCCCCCGTAT<br>CTCTCCTACGGCGCGACAATCAATTTCGTCGACTAGAAATGATTATTCATAA<br>CA
MT613062	>HvarSat16-87<br>AGAATTTATATGTTTTTTGAGATCTCCTTTAATATTGGAAGAATACTCACACA<br>AATGACATTTATGTTTCTTCAAATTCAAATGAAC
MT613063	>HvarSat17-176<br>CACTCTCGACTTGATCGCATAATAATTTTTTTTTGTAGGTTTGTGATGTCCGG<br>CCAAGAACTTTGAGTGTTGTTTTCGAAAGATCATTTTATTATGTACTTTTTTTA<br>AAACTTTCGTTTCATTCTATGAACCCTAGACCCCCAGCTCCAAGAGTCGAAA<br>AATGCCGTCTTTGGCGT
MT613064	>HvarSat18-191<br>AGCGAGGTCACCGCTCTGTCACAACCCCTAATCTTGTCGAACGTCTACACCC<br>CGAGGTTGGAGCTATCATTGAGTTGATACTCCATCGTCCCTCAAGTGGAATC<br>AGTCGTGGGAGTTCCTAATTGCGAGCAACTCTTATTAGGAGCAACCAGGCC

	AGGTTCCTCATCTTGAGGAAAAGGGTAACCTTTCCG
MT613065	>HvarSat19-143<br>TCGAAAATCGAGCGTGAATATTAAGGGTTGAAATTTCAACCCCTGAACCCC<br>ATCGCAAGTCAAAATTTTTTTTCGATATCGAATAATTATGTCACGAAGAATA<br>TTTCTGCCAAGTTTTATGATGCAGGTATTACGTTTTCCCT
MT613066	>HvarSat20-141<br>TTTTTTCAACCCTTAACTTTGAAAAATTCATATCTCGGAAACTATTCTCCGGA<br>GCACATACAGACTTCGTATGTTGTTTCATTATGAGGAAACCAATGATCCCTG<br>AAAATATGATCGACTTCCCGAAGGAGTACGGGGGTG
MT613067	>HvarSat21-152<br>TGGTTCGCGCATTTCGAACCGAGATATAAGCAAAAAAATGTCAAAATGACA<br>GTTTTTTCAATTTCCTGTCTCAAATAACGTGCCCCAAATTGATTTGAAAGAAG<br>AAATCAATTCACCAAAGAGCGATGCCCACTCTCGTGAAAAGATTTTCG
MT613068	>HvarSat22-145<br>TATAGATTTGGAAAAATTGGAAATTCAATTTTCCGGGCCAACTTATGTGCAG<br>CCCGGAAAACCCAGCATTTTTCATCGATATTTCGAAAATTGAAAATGCTAGC<br>GAAATGCTGGCGAATGCTGAAGAAAAAACTGAAAAATCTCT
MT613069	>HvarSat23-378<br>GAGATGAAAGATGAGAAGAAACCGAAAACGCCTTCATCTGATAAAACGCC<br>AGAACTTTTCGAAAAAGTGAAGAAATCACCTCAGTCTCCAGCTGATCGAGT<br>ACCGGGAGTAACTGAAAAAGTACCCAGTTCTGTTGAGACGCCTAAATCGGT<br>TTCACCTGTAGATGTTCTTGAACACAAAGTAATTACCGAGTCAGTAGAAATG<br>AAAGAAACAATTATCAAAAAATCACCAGAGTCTATCGAGAAACCTAGTCA<br>CGACATTCCTTCTCCCAAAACTGAACATATTGTTGATTCAACTGAAAAGAAG<br>AAGGAACCCATGACTCCGACAAAGGATCATGTTGAAAAACTACCATTGAAA<br>CCGGTTGATCATGTCTCAGAA
MT613070	>HvarSat24-105<br>GATACTCGTCGAGGCAGTCGACCCAATGCTAGATGTTACAGTTGTGTCTGCT<br>TCAGATGTGCTACTTTCAGTAGTTGGTTCAGATGGTTCTGTTGTATCTGGTGC
MT613071	>HvarSat25-150<br>ATGAAATATCGTTAAAATACGTATCGAAGAATAGGAAAAATCATATTTCAT<br>CTTGACAGTCATTTGCGAAATAGTTGAGAAAAATTTTTGAAAATTTCATTTTC<br>AGGGGGTAATTTCACTAAGAAGGCAGGGGGTGCAAGAAATTTTTTC
MT613072	>HvarSat26-164<br>CCACTGAAAAAATCGTTCACAAAAAAGTAATATTTATAGGGAAAAAAAAA<br>TTTTTTCTATTGTCGGAAACCGTGGCTGGCTCGAAATTTCGATTTTCCGATTC<br>CCAACCATGGGTCAACTCTGTTTGAAAATTTTTTCTGTTAGAGTTCATCAATA<br>CCAACAAT
MT613073	>HvarSat27-41<br>AGATGAAGATTCAACAATAGCCAAGCAGAACAAATAGAAAT
MT613074	>HvarSat28-57<br>GAGAGTTGAAACCAAGAGGGTTTCTTCGGCTTGCTCGAGGAAGTAGTGTCC<br>GGGGAC
MT613075	>HvarSat29-169<br>TTTTCATTACAATTCAATTCTGGTTAGCGTTTCCAGCAGGAGAATCTATGCAA<br>TATAAAAACATATAACGTTATTGATTTCATCTACATTCGTATCTCTTTTAATT<br>CAGTTCAATTCAGGTTAGAAATTTGAAGACTTGCTTCCAATAGGAGAATCTA<br>TGCAATATGGA
